# Disease-specific patterns of glymphatic-related dysfunction between multiple sclerosis and neuromyelitis optica spectrum disorder

**DOI:** 10.3389/fimmu.2026.1917373

**Published:** 2026-09-18

**Authors:** Xiaoya Chen, Kai Zhang, Xiaojuan Dong, Xiaohui Zhang, Zhuowei Shi, Zichun Yan, Qiyuan Zhu, Yuling Peng, Yongliang Han, Yongmei Li

**Affiliations:** Department of Radiology, The First Affiliated Hospital of Chongqing Medical University, Chongqing, China

**Keywords:** diffusion tensor image analysis along the perivascular space, glymphatic system, multiple sclerosis, neuromyelitis optica spectrum disorder, periventricular diffusivity

## Abstract

**Background:**

Glymphatic dysfunction has been reported in multiple sclerosis (MS) and neuromyelitis optica spectrum disorder (NMOSD), yet disease-specific patterns of glymphatic-related impairment and their structural and cognitive consequences remain poorly characterized.

**Methods:**

This study enrolled 196 MS patients, 88 NMOSD patients, and 108 healthy controls (HC). Glymphatic-related function was assessed using diffusion tensor image analysis along the perivascular space (DTI-ALPS) and periventricular diffusivity (PVeD). Between-group differences, partial correlations with structural and clinical outcomes, AQP4 subgroup comparisons, mediation analyses, and subcortical atrophy-based subgroup analyses were performed to characterize disease-specific glymphatic-related impairment patterns.

**Results:**

MS showed more extensive glymphatic-related impairment than NMOSD, with significant reductions in both DTI-ALPS and PVeD compared to HC (all p<0.001). PVeD was significantly reduced in NMOSD compared to HC in both cohorts (p ≤ 0.008), whereas DTI-ALPS differences in NMOSD relative to HC were inconsistent across cohorts, reaching significance only in the GE replication cohort. In MS, PVeD showed widespread structural and cognitive associations; in NMOSD, associations were restricted to ventricular and choroid plexus volumes. A parallel multiple mediation model showed that subcortical gray matter atrophy, but not lesion volume, independently mediated the association between PVeD and Symbol Digit Modalities Test performance in MS, a pattern absent in NMOSD. Stratified subgroup analysis showed a pattern consistent with a glymphatic-cognitive association specific to patients with greater subcortical atrophy in MS, although formal interaction tests did not reach statistical significance; no such association was observed in either atrophy subgroup in NMOSD. AQP4-seropositive, but not seronegative, NMOSD patients showed significantly lower glymphatic-related metrics than HC (p<0.001); direct comparison between the two subgroups was not significant.

**Conclusion:**

Glymphatic-related dysfunction exhibits distinct disease-specific patterns in MS and NMOSD, with PVeD showing broader sensitivity across both cohorts. These findings support PVeD as a promising complementary glymphatic-related diffusion marker in inflammatory demyelinating disease, though causal and disease-mechanistic interpretations await validation in longitudinal and mechanistic studies.

## Introduction

Multiple sclerosis (MS) and neuromyelitis optica spectrum disorder (NMOSD) are chronic inflammatory demyelinating diseases of the central nervous system characterized by distinct immunopathological mechanisms (1). MS is primarily driven by immune-mediated demyelination and neurodegeneration, whereas NMOSD is commonly associated with antibodies against aquaporin-4 (AQP4), a water channel highly expressed on astrocytic endfeet (2, 3). Despite these differences, both disorders exhibit substantial neurological disability and brain tissue damage that cannot be fully explained by focal inflammatory lesions alone (4).

The glymphatic system has emerged as an important pathway for cerebrospinal fluid (CSF)-interstitial fluid (ISF) exchange and metabolic waste clearance in the brain (5, 6). Growing MRI evidence indicates that glymphatic abnormalities are present in both MS and NMOSD. In MS, impaired glymphatic function has been consistently associated with greater disability, lesion burden, brain atrophy, and disease progression have been linked to progressive disease phenotypes and future disability accumulation (7, 8). In NMOSD, enlarged perivascular spaces and alterations in glymphatic-related imaging markers have also been reported, with significant associations with disability, AQP4-antibody titers, blood-brain barrier dysfunction, and ventricular enlargement (9, 10). These findings suggest that glymphatic-related dysfunction may represent a shared pathological feature across inflammatory demyelinating diseases.

Diffusion tensor image analysis along the perivascular space (DTI-ALPS) has been proposed as a non-invasive MRI marker of glymphatic-related function and has been widely applied in neurological disorders (11), including Alzheimer’s disease, Parkinson’s disease and other neurological disorders (12–15). However, findings regarding DTI-ALPS in inflammatory demyelinating disease have been inconsistent: a recent comparative study using Bayesian hypothesis testing reported no significant differences in DTI-ALPS or free-water metrics between MS and NMOSD (16), and methodological work has shown that the DTI-ALPS index may be influenced by white matter microstructure and fiber geometry, which may limiting its specificity and reproducibility as a glymphatic-related biomarker in diseases marked by extensive white matter pathology (17, 18). These inconsistencies suggest a need for a complementary diffusion-based indicator, less dependent on fiber orientation, to help clarify whether disease-specific glymphatic-related alterations exist in MS and NMOSD.

Periventricular diffusivity (PVeD) is a recently proposed diffusion-based metric that characterizes diffusivity within periventricular white matter regions closely related to CSF-ISF exchange, using a fully automated delineation strategy intended to reduce ROI dependency and operator variability (19). Like DTI-ALPS, PVeD is an indirect, diffusion-derived proxy rather than a direct measure of glymphatic clearance or CSF-ISF flow. PVeD has shown promising associations with glymphatic-related dysfunction, neurodegeneration, and cognitive decline in Alzheimer’s disease, but it has not yet been examined in MS or NMOSD, nor has it been validated against established glymphatic reference methods or against DTI-ALPS in inflammatory demyelinating disease. Whether PVeD offers complementary, and potentially more sensitive, index relative to DTI-ALPS for detecting disease-specific glymphatic-related alterations in MS and NMOSD is therefore an open question that the present study was designed to address.

The present study therefore aimed to: (1) characterize disease-specific patterns of glymphatic-related dysfunction in MS and NMOSD using both DTI-ALPS and PVeD across two independent scanner cohorts; (2) examine associations between glymphatic-related metrics and structural neuroimaging and cognitive outcomes in each disease; (3) investigate whether AQP4 antibody status modulates glymphatic-related function within NMOSD; and (4) determine whether subcortical gray matter atrophy moderates the relationship between glymphatic-related dysfunction and cognitive impairment in MS.

## Materials and methods

### Participants

This study utilized two independent cohorts acquired on different MRI platforms. AQP4-IgG serostatus was determined from clinical laboratory records obtained during hospitalization; NMOSD patients were classified as seropositive (NMOSD+) or seronegative (NMOSD-). The Siemens primary cohort comprised 55 HC, 150 MS patients, and 49 NMOSD patients (38 NMOSD+, 11 NMOSD-). The GE replication cohort comprised 53 HC, 46 MS patients, and 39 NMOSD patients (29 NMOSD+, 10 NMOSD-). MS was diagnosed according to the 2017 McDonald criteria (20) and NMOSD according to the 2015 International Consensus diagnostic criteria (21). All MS patients had the relapsing-remitting subtype, and none showed gadolinium-enhancing lesions at the time of scanning. In the Siemens cohort, 43.3% of MS patients were receiving disease-modifying therapy (DMT) at the time of scanning (most commonly teriflunomide and siponimod), while 56.7% were untreated; no MS patients in the GE cohort were receiving DMT at the time of scanning. The study was approved by the Institutional Review Board of the First Affiliated Hospital of Chongqing Medical University, and all participants provided written informed consent.

### Clinical assessment

Demographic and neuropsychological data, including the Digit Span Test (DST), Symbol Digit Modalities Test (SDMT), Mini-Mental State Examination (MMSE), and Montreal Cognitive Assessment (MoCA), were recorded for all participants. Clinical data, including disease duration and Expanded Disability Status Scale (EDSS) scores, were also recorded for patients.

### MR image acquisition

All participants underwent brain MRI scanning on one of two 3.0 T scanners. Scanner 1 (MAGNETOM Skyra, Siemens, Erlangen, Germany) was equipped with a 32-channel phased-array standard head coil, and Scanner 2 (GE Healthcare, Milwaukee, USA) was equipped with an 8-channel phased-array standard head coil. Scan protocols were as follows:

Siemens: (i) sagittal 3-dimensional (3D) T1w magnetization-prepared rapid gradient echo [repetition time (TR) = 2300 ms, echo-time (TE) = 2.26 ms, inversion time (TI) = 900 ms, 192 slices, field of view (FOV) = 256 mm, voxel size = 1.0 × 1.0 × 1.0 mm3], (ii) sagittal 3D fluid attenuated inversion recovery (FLAIR) (TR = 5000 ms, TE = 388 ms, TI = 1800 ms, 192 slice, FOV = 256 mm, voxel size = 0.5 × 0.5 ×1 mm3), (iii) diffusion kurtosis imaging (DKI) sequence (TR = 5000 ms, TE = 97 ms, 72 slices, FOV = 220 mm, voxel size = 1.7 × 1.7 × 2.0 mm3, Partial-Fourier = 6/8, integrated parallel acquisition techniques acceleration factor = 2, and three b values (0, 1,000, and 2,000 s/mm2) with diffusion encoding in 60 directions.

GE: 1) A high-resolution three-dimensional (3D) T1-weighted image was acquired with repetition time (TR) 8300 ms, echo time (TE) 3.3 ms, isotropic voxels of dimension 1 mm × 1 mm × 1mm, 156 slices, Field of View (FOV) 256 mm × 256 mm. 2) A high-resolution Diffusion Tensor Imaging (DTI) was performed with TR 5700 ms, TE 86 ms, voxel size 2.5 mm × 2.5 mm × 2.3 mm, 53 slices, 34 diffusion directions and b-values of 0 and 1000s/mm2.

### MRI data processing and analysis

#### Measurement of T_2_WI lesion volume

The Lesion Segmentation Tool (LST) (22) and Statistical Parametric Mapping 12 (SPM) software (https://www.fil.ion.ucl.ac.uk/spm/) were used to automatically measure the total volume of lesions in patients using 3DT1 and T2-FLAIR images.

#### 3DT_1_ data processing

The recon-all code in FreeSurfer 6.0 software (https://surfer.nmr.mgh.harvard.edu/fswiki/) was used to segment brain tissue. Eight bilateral deep gray matter structures were segmented: thalamus, caudate nucleus, putamen, globus pallidus, hippocampus, amygdala, nucleus accumbens, and ventral diencephalon. Ventricular volumes (lateral ventricles and third ventricle) and total CSF volume were extracted for analysis. Choroid plexus segmentation and volumetric measurements were obtained using a fully automated deep learning-based method applied to 3D T1-weighted image (https://github.com/hettk/chp_seg).

#### DTI processing

##### Calculation of DTI-ALPS

DTI-ALPS was computed by manually delineating four bilateral ROIs in the projection and association fiber areas, following established procedures (11). The index was calculated as: ALPS index = mean (Dxxproj, Dxxassoci)/mean (Dyyproj, Dzzassoci). Left and right indices were derived separately by two independent raters (ZK and DX in the Siemens cohort; CX and PY in the GE cohort).

##### Calculation of PVeD

PVeD was computed following the framework of Chen et al. (19), adapted for demyelinating diseases. Voxel-wise transverse tensor ratio (TTR = Dxx/|D|_2_) was calculated under the anatomical assumption that deep medullary veins run predominantly along the left-right axis. The periventricular area was defined automatically by registering a lateral ventricle pre-mask from MNI space to each subject’s diffusion space, followed by directional dilation constrained to periventricular white matter. PVeD was defined as the median TTR within the periventricular area; full pipeline details, including the lesion exclusion sensitivity analysis, are provided in **Supplementary Methods**.

The schematic illustrations of DTI-ALPS and PVeD are presented in **Figure 1**.

**Figure 1:**
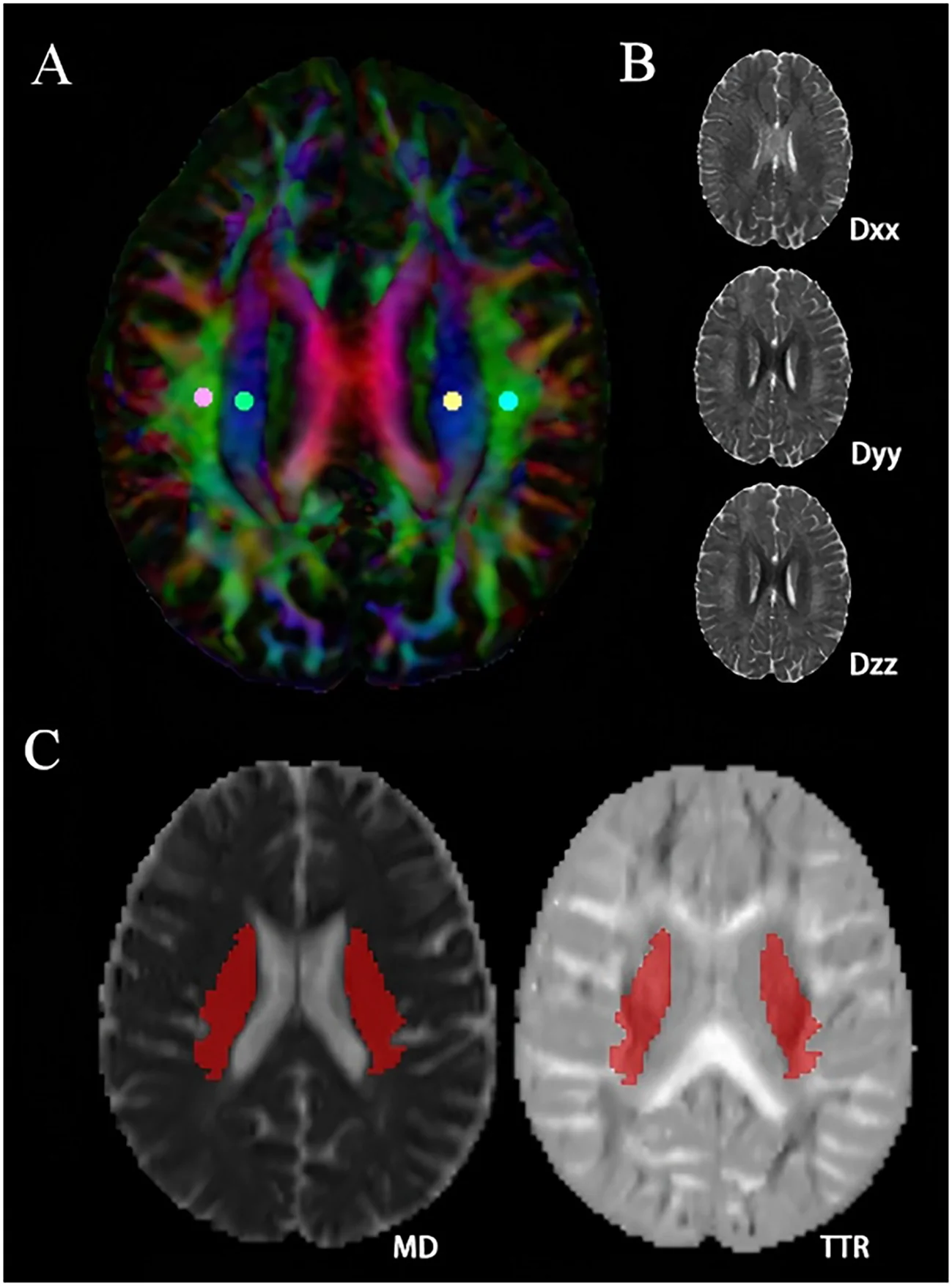
Illustration of DTI-ALPS and PVeD calculation. **(A)** Fractional anisotropy (FA) maps at the level of the lateral ventricle body, overlaid with predefined regions of interest (ROIs) for projection fibers and association fibers used for DTI-ALPS calculation. **(B)** Corresponding directional diffusivity maps (Dxx, Dyy, Dzz) derived from the diffusion tensor, from which diffusivity values along the x-, y-, and z-axes were extracted within each ROI. The DTI-ALPS index was computed as:ALPS index = mean (Dxxproj, Dxxassoci)/ mean (Dyyproj, Dzzassoci). **(C)** ROI masks used for periventricular diffusivity (PVeD) calculation, overlaid on mean diffusivity (MD) and tensor trace (TTR) maps. PVeD was defined as the median transverse tensor ratio (TTR = Dxx / |D|_2_) within the periventricular area, reflecting diffusivity along the predominant perivascular space direction.

### Statistical analysis

Between-group differences were assessed using ANCOVA (covariates: age, sex), with analyses conducted separately in each cohort. Effect sizes were quantified using Cohen’s d (absolute standardized mean difference), and mean Cohen’s d values were calculated by averaging effect sizes across all pairwise group comparisons within each cohort to compare the overall sensitivity of PVeD versus DTI-ALPS. ComBat harmonization was applied to DTI-ALPS and PVeD values (scanner as batch variable; group, age, and sex as protected covariates), and these effect sizes were recalculated using harmonized values for comparison before versus after harmonization. A lesion exclusion sensitivity analysis was additionally performed to determine whether PVeD group differences were attributable to lesion-related demyelination rather than glymphatic-related diffusivity, in which visible periventricular lesion voxels were excluded from the PVeD region of interest in MS and NMOSD patients, PVeD was recalculated, and the intraclass correlation coefficient (ICC(2,1)) and Bland-Altman analysis were used to assess agreement between original and lesion-excluded values.

Partial Spearman correlations, adjusted for age and sex, were computed between glymphatic metrics and neuroimaging and clinical outcomes, with FDR correction applied. Inter-rater reliability of DTI-ALPS was assessed using ICC. A sensitivity analysis additionally adjusted for mean diffusivity. AQP4 subgroup comparisons combined both cohorts, with scanner identity included as an additional covariate.

Mediation analyses were conducted in the Siemens MS cohort, in which complete clinical data were available. SDMT was selected as the primary outcome. Two parallel models were tested with PVeD as the independent variable: (1) subcortical gray matter composite score as the mediator, and (2) white matter lesion volume as the mediator, both with SDMT as the outcome, estimated using bias-corrected bootstrap confidence intervals (5000 iterations). Identical models were estimated in the NMOSD group for disease-specific comparison. To further characterize the structural context of glymphatic-cognitive coupling, MS and NMOSD patients were stratified into high and low subcortical atrophy subgroups based on the median composite score of 14 bilateral subcortical structures. Between-group differences in glymphatic-related metrics and partial correlations between glymphatic-related metrics and SDMT were examined within each atrophy subgroup, and the moderating effect of subcortical atrophy on the glymphatic-cognitive association was assessed using interaction terms in linear regression models.

## Results

### Demographic, clinical and neuropsychological variables

Demographic and clinical characteristics are summarized in **Table 1**. In both cohorts, groups were well matched for age and disease duration (all p > 0.05). A significant sex imbalance was observed in the GE cohort (χ² = 13.447, p = 0.001), which was addressed by including sex as a covariate in all analyses. EDSS was significantly higher in NMOSD than MS in the Siemens cohort (2.31 ± 1.72 vs. 1.36 ± 1.64, p < 0.001) but not in the GE cohort (p = 0.209). In the Siemens cohort, both MS and NMOSD patients showed significantly lower SDMT and MoCA scores than HC (all p < 0.05), with NMOSD patients showing lower MoCA scores than MS patients (p < 0.05).

**Table 1:** Demographic and clinical characteristics of study participants.

Variable	Siemens cohort (primary)				GE cohort (replication)			
	HC (n=55)	MS (n=150)	NMOSD (n=49)	p value	HC (n=53)	MS (n=46)	NMOSD (n=39)	p value
Demographics								
Age (years)	36.60 ± 9.86	34.08 ± 9.76	36.16 ± 11.67	0.2	37.30 ± 14.45	40.85 ± 13.67	41.05 ± 14.30	0.342
Sex (F/M)	39F / 16M	112F / 38M	43F / 6M	0.097	29F / 24M	28F / 18M	35F / 4M	0.001
Clinical (patients only)								
Disease duration (years)	—	4.87 ± 5.16	5.26 ± 5.42	0.649	—	7.40 ± 5.51	5.32 ± 5.80	0.102
EDSS	—	1.36 ± 1.64 ^c^	2.31 ± 1.72 ^c^	<0.001	—	2.96 ± 1.46	3.40 ± 1.06	0.209
AQP4+ / AQP4−	—	—	38+ / 11−	—	—	—	29+ / 10−	—
DMT status (treated/untreated)	—	43.3%/56.7%	—	—	—	—	—	—
Neuropsychological (Siemens only)								
DST	15.13 ± 2.52	13.92 ± 2.50 ^a^	14.20 ± 3.03	0.015	N/A	N/A	N/A	N/A
SDMT	54.71 ± 12.49	48.11 ± 16.20 ^a^	44.74 ± 15.43 ^b^	0.003	N/A	N/A	N/A	N/A
MMSE	29.11 ± 1.08	28.55 ± 1.79 ^a^	27.80 ± 3.93 ^b^	0.013	N/A	N/A	N/A	N/A
MoCA	27.29 ± 2.36	25.65 ± 3.52 ^ac^	24.10 ± 4.60 ^bc^	<0.001	N/A	N/A	N/A	N/A

Values presented as mean ± SD or n (F/M). Superscripts denote significant post-hoc pairwise comparisons: a = p < 0.05, HC vs MS; b = p < 0.05, HC vs NMOSD; c = p < 0.05, MS vs NMOSD. p values: age = one-way ANOVA; sex = chi-square test; disease duration and EDSS = independent-samples t-test (MS vs NMOSD); neuropsychological scales = one-way ANOVA. Clinical scales available in Siemens cohort only. *p<0.05; **p<0.01; ***p<0.001.

### Group differences in glymphatic metrics

In the Siemens primary cohort, MS patients exhibited significantly lower DTI-ALPS and PVeD compared to HC (all p < 0.001). NMOSD patients showed significantly lower PVeD compared to HC (p = 0.004), whereas DTI-ALPS did not differ significantly between NMOSD and HC (p > 0.05). MS patients also showed significantly lower PVeD and DTI-ALPS than NMOSD (both p < 0.05). In the GE replication cohort, MS showed significantly lower DTI-ALPS and PVeD compared to HC (all p < 0.001). NMOSD showed significantly lower DTI-ALPS and PVeD compared to HC (both p < 0.01). MS showed significantly lower DTI-ALPS and lower PVeD Mean than NMOSD (both p < 0.05). Exact effect sizes, confidence intervals, and p-values for all comparisons are shown in **Figure 2**; detailed inter-group differences for the left and right sides are presented in **Supplementary Figure 1**.

**Figure 2:**
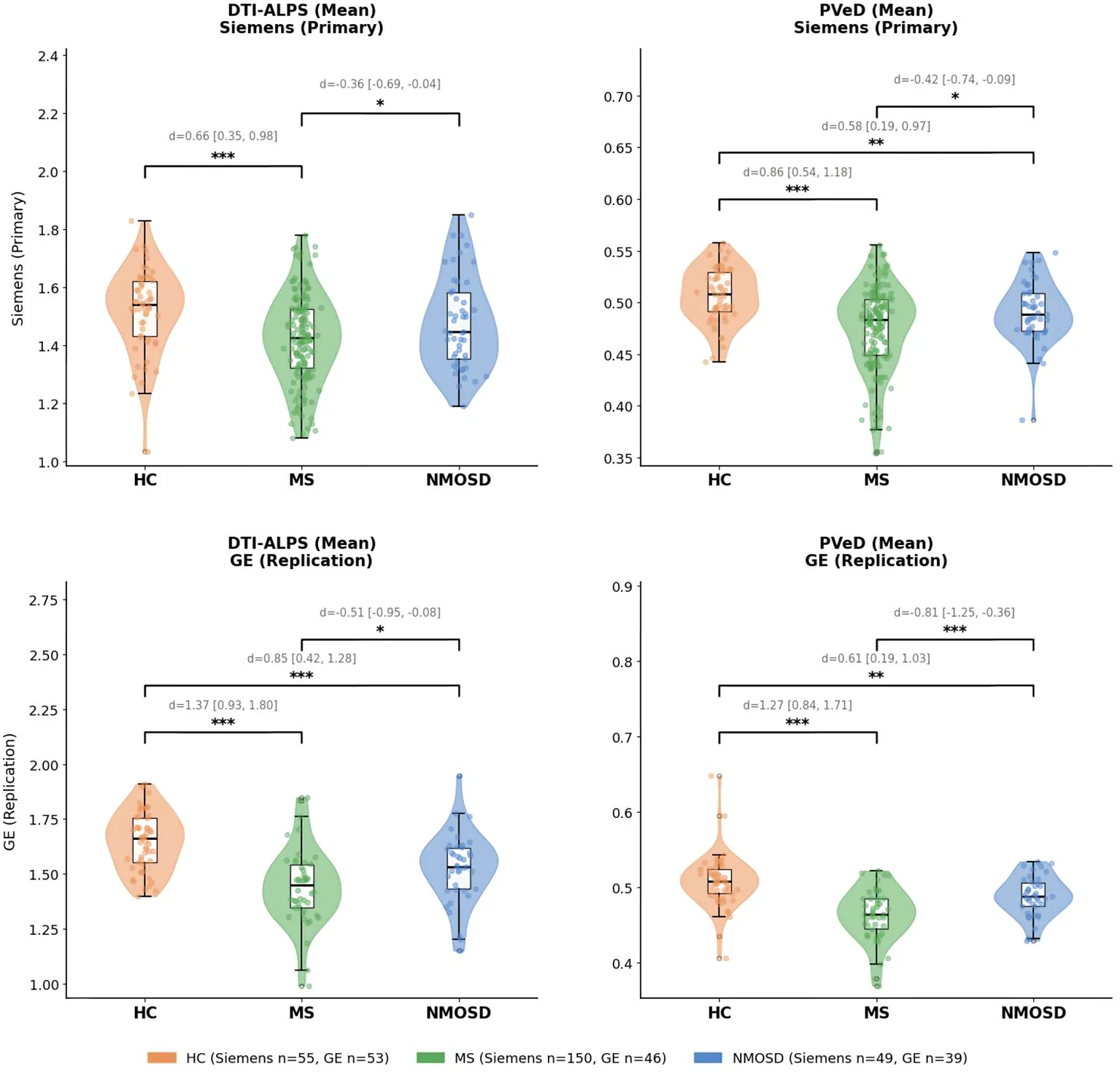
Group comparisons of DTI-ALPS and PVeD (Mean) across HC, MS, and NMOSD. Violin plots with embedded box plots and individual data points show the distribution of DTI-ALPS (Mean) and PVeD (Mean) in the Siemens primary cohort (top row) and GE replication cohort (bottom row). Box plots indicate the median and interquartile range; whiskers extend to 1.5 times the interquartile range. Between-group differences were assessed using ANCOVA with age and sex as covariates. For each significant pairwise comparison, Cohen's d and its 95% confidence interval are labeled above the corresponding significance bracket, with asterisks denoting significance level (*p < 0.05, **p < 0.01, ***p < 0.001); comparisons not reaching significance are not bracketed. Full right- and left-hemisphere results are provided in **Supplementary Figure S1** and **Supplementary Table S1**. HC, healthy controls; MS, multiple sclerosis; NMOSD, neuromyelitis optica spectrum disorder.

Across all pairwise group comparisons, PVeD demonstrated a larger mean effect size than DTI-ALPS (mean Cohen’s d = 0.729 vs 0.611; **Supplementary Table 1**).

A cross-platform harmonization analysis using ComBat was performed; effect sizes changed minimally after harmonization (mean absolute change in Cohen’s d = 0.009; range 0.002-0.030; **Supplementary Table 2**).

ICC analysis of DTI-ALPS demonstrated moderate inter-rater reliability in both the Siemens cohort (ICC = 0.507, 95% CI [0.35, 0.62], p < 0.001) and the GE cohort (ICC = 0.524, 95% CI [0.40, 0.62], p < 0.001).

### Lesion exclusion sensitivity analysis

To determine whether PVeD group differences reflect lesion-related demyelination rather than glymphatic-related dysfunction, PVeD was recalculated after excluding visible periventricular lesion voxels. Agreement between original and lesion-excluded values was excellent in both cohorts (ICC(2,1) = 0.991; **Supplementary Figure 2**), and between-group effect sizes for mean PVeD remained largely unchanged (**Supplementary Table 3**). The HC-versus-NMOSD difference in PVeD Left, however, became non-significant after lesion exclusion in both cohorts. A parallel sensitivity analysis using lesion-excluded PVeD values showed a highly consistent pattern of significant correlations with structural and clinical outcomes in the MS group (Siemens: 23 vs. 23; GE: 16 vs. 14 significant correlations; **Supplementary Figure 3**).

### Partial correlation analysis

The associations between glymphatic-related metrics and neuroimaging and clinical measures in MS and NMOSD are shown in **Figure 3**.

**Figure 3:**
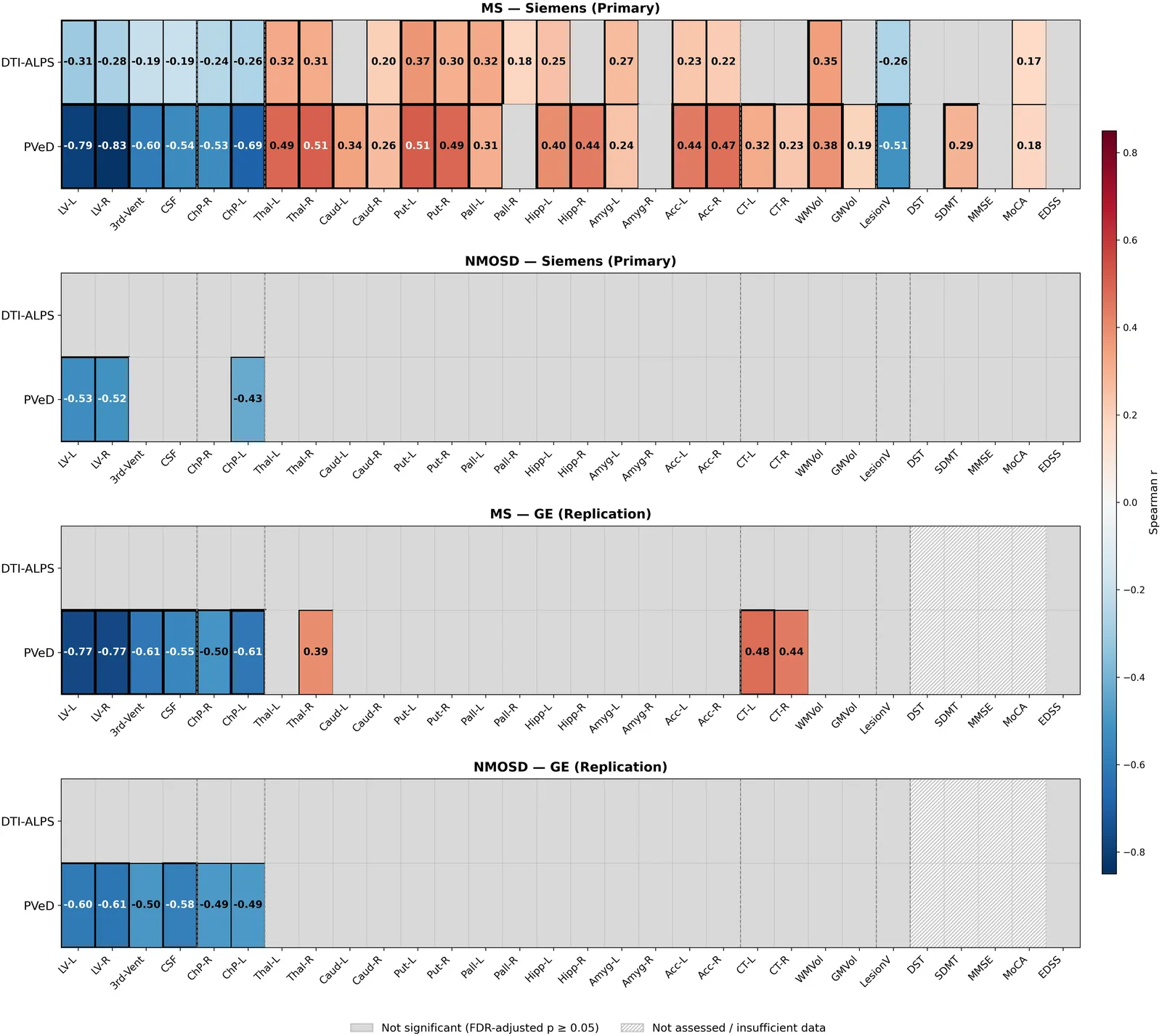
Partial correlation heatmaps of glymphatic-related indices (Mean) with neuroimaging and clinical measures. Partial Spearman correlations between DTI-ALPS (Mean) and PVeD (Mean) and structural neuroimaging measures (ventricular volumes, choroid plexus volumes, subcortical gray matter volumes, cortical thickness, white matter and gray matter volumes, lesion volume) and clinical measures (DST, SDMT, MMSE, MoCA, EDSS) are shown in four panels: MS and NMOSD groups, each in the Siemens primary cohort and GE replication cohort. All correlations were adjusted for age and sex. Multiple comparisons were corrected using the Benjamini-Hochberg FDR procedure (p_FDR < 0.05). Light gray cells indicate correlations tested but not significant after FDR correction; hatched white cells indicate correlations that could not be assessed due to missing data, including DST, SDMT, MMSE, and MoCA in the GE cohort, where cognitive assessments were not collected. Border thickness indicates significance level: thin = p_FDR < 0.05; medium = p_FDR < 0.01; thick = p_FDR < 0.001. Full right- and left-hemisphere results are provided in Supplementary **Supplementary Figure S1**. LV, lateral ventricle; ChP, choroid plexus; Thal, thalamus; Caud, caudate; Put, putamen; Pall, pallidum; Hipp, hippocampus; Amyg, amygdala; Acc, accumbens; CT, cortical thickness; WMVol, white matter volume; GMVol, gray matter volume; LesionV, lesion volume; L, left; R, right.

In the MS group of the Siemens cohort, PVeD demonstrated extensive correlations with structural neuroimaging measures, including strong negative correlations with lateral ventricular volumes (LV-L: r = -0.79; LV-R: r = -0.83) and choroid plexus volumes (ChP-R: r = -0.53; ChP-L: r = -0.69), positive correlations with subcortical gray matter volumes (r = 0.24-0.51), cortical thickness (CT-L: r = 0.32; CT-R: r = 0.23), and white matter volume (WMVol: r = 0.38; GMVol: r = 0.19), and negative correlations with lesion volume (r = -0.51). PVeD was also significantly associated with SDMT (r = 0.29) and MoCA (r = 0.18). DTI-ALPS showed significant but generally weaker associations with similar structures in the Siemens MS group (e.g., lateral ventricular volumes: r = -0.28 to -0.31; subcortical gray matter: r = 0.18-0.37), including a weak association with MoCA (r = 0.17) but not SDMT. In the GE replication cohort, PVeD associations were largely replicated across ventricular (LV-L/R: r = -0.77), choroid plexus (ChP-R: r = -0.50; ChP-L: r = -0.61), and cortical thickness measures (CT-L: r = 0.48; CT-R: r = 0.44), with an additional positive correlation with right thalamic volume (Thal-R: r = 0.39), whereas no DTI-ALPS correlations reached significance in this cohort.

In contrast, the NMOSD group showed markedly more restricted associations. In the Siemens cohort, significant correlations were observed only between PVeD and ventricular volumes (LV-L: r = -0.53; LV-R: r = -0.52) and choroid plexus volume (ChP-L: r = -0.43), with no significant DTI-ALPS correlations. In the GE replication cohort, PVeD showed a broader pattern of significant correlations with ventricular (LV-L: r = -0.60; LV-R: r = -0.61), periventricular (3rd-Vent: r = -0.50; CSF: r = -0.58), and choroid plexus volumes (ChP-R: r = -0.49; ChP-L: r = -0.49), again with no significant DTI-ALPS correlations.

Sensitivity analyses confirmed that PVeD associations with structural outcomes were largely independent of mean diffusivity, while DTI-ALPS associations were substantially attenuated after MD adjustment, as shown in **Supplementary Table 4**.

### Mediation analysis

Two separate single-mediator models were first tested in the Siemens MS cohort with SDMT as the outcome (**Supplementary Figure 4**). Subcortical gray matter atrophy significantly mediated the PVeD-SDMT association (indirect effect = 0198, 95% CI [0.094, 0.305], p < 0.001, Proportion Mediated = 70.1%), as did lesion volume (indirect effect = 0.130, 95% CI [0.050, 0.237], p = 0.006, Proportion Mediated = 44.2%). Neither model was significant in NMOSD (p = 0.280 and 0.158).

As the two mediators were correlated (r = -0.62), we then conducted a parallel multiple mediation model including both simultaneously (**Figure 4**). The specific indirect effect via subcortical gray matter atrophy remained significant (0.192, 95% CI [0.089, 0.294], p < 0.001), while the effect via lesion volume did not (0.040, 95% CI [-0.037, 0.132], p = 0.345). The total indirect effect remained significant (0.233, 95% CI [0.123, 0.353], p < 0.001, Proportion Mediated = 80.6%). Mediators were weakly correlated in NMOSD (r = -0.08), and no indirect effects were significant (all p > 0.11).

**Figure 4:**
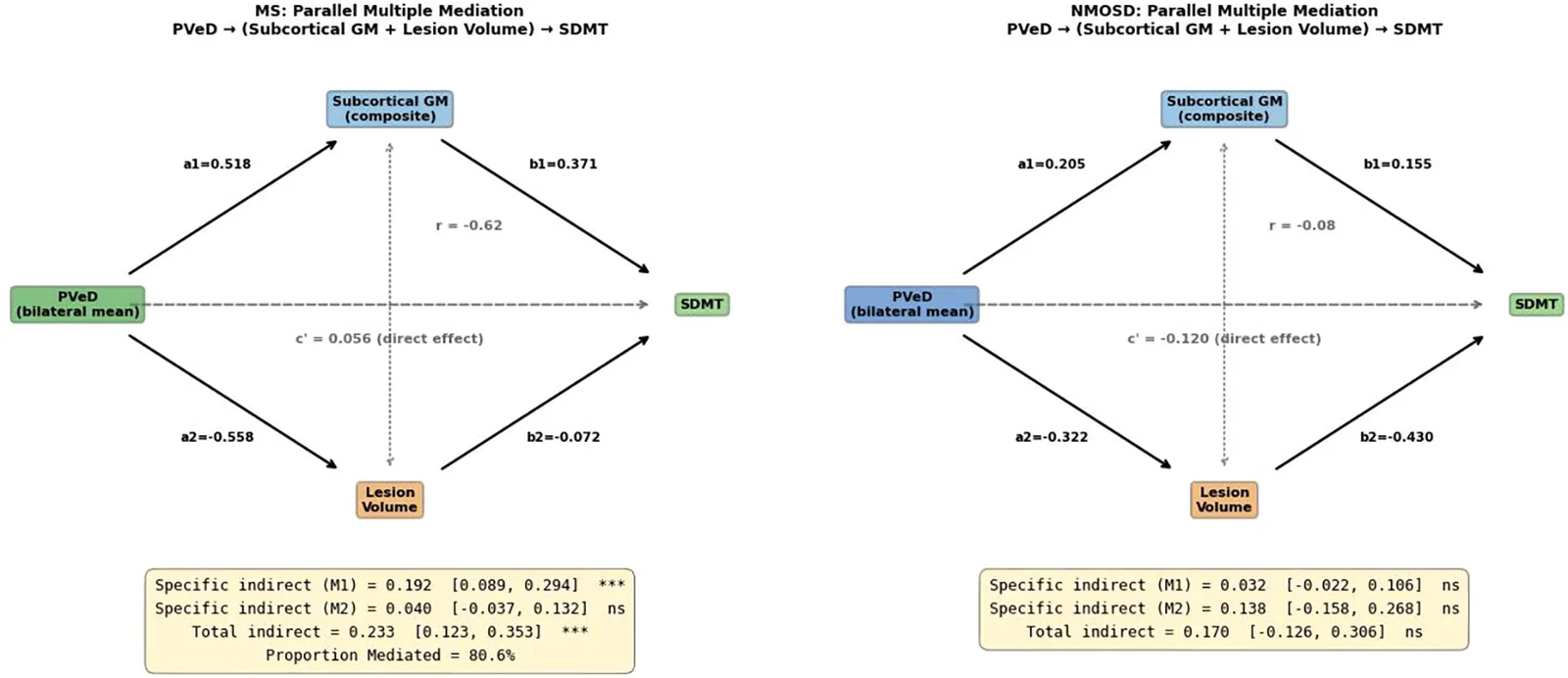
Parallel multiple mediation analysis linking PVeD to cognitive performance in MS and NMOSD. Path diagrams showing parallel multiple mediation models testing subcortical gray matter atrophy (composite score) and lesion volume as simultaneous mediators of the association between PVeD (bilateral mean) and SDMT in the Siemens MS cohort (left) and NMOSD cohort (right; control analysis). Path coefficients (a1, a2, b1, b2) and the direct effect (c') are standardized regression coefficients adjusted for age and sex. The dotted line between mediators indicates their bivariate correlation (r). Specific indirect effects, the total indirect effect, and 95% bootstrap confidence intervals (5,000 iterations) are shown in the boxed panel below each diagram (***p < 0.001; ns, not significant).

### Cognitive domain specificity analysis

To determine whether the PVeD-SDMT association reflected a specific relationship with information processing speed rather than overall disease severity, we conducted two supplementary analyses in the Siemens MS cohort. First, the partial correlation between PVeD and SDMT remained significant after additionally controlling for EDSS (r = 0.271, p < 0.001, versus r = 0.287, p < 0.001 without EDSS). Second, using Steiger’s Z tests to compare dependent correlations, the PVeD-SDMT association was significantly stronger than the PVeD-DST (z = 2.61, p = 0.009) and PVeD-MMSE (z = 2.68, p = 0.007) associations, but did not differ significantly from the PVeD-MoCA association (z = 1.36, p = 0.173; **Supplementary Table 5**).

### Atrophy subgroup analysis

As shown in **Figure 5**, both PVeD and DTI-ALPS were significantly lower in the high atrophy subgroup compared to the low atrophy subgroup in MS (PVeD: p < 0.001; DTI-ALPS: p < 0.05). Stratified partial correlation analyses showed that both PVeD and DTI-ALPS were significantly associated with SDMT in the high atrophy subgroup (PVeD: r = 0.52, p < 0.001; DTI-ALPS: r = 0.44, p < 0.001), whereas no significant associations were observed in the low atrophy subgroup (PVeD: r = 0.07, p = 0.578; DTI-ALPS: r = -0.06, p = 0.614). However, formal interaction models (SDMT ~ metric × atrophy status + age + sex) did not confirm a statistically significant moderating effect of atrophy status for either PVeD (β = -0.076, 95% CI [-0.188, 0.036], p = 0.181) or DTI-ALPS (β = -0.103, 95% CI [-0.224, 0.017], p = 0.093).

**Figure 5:**
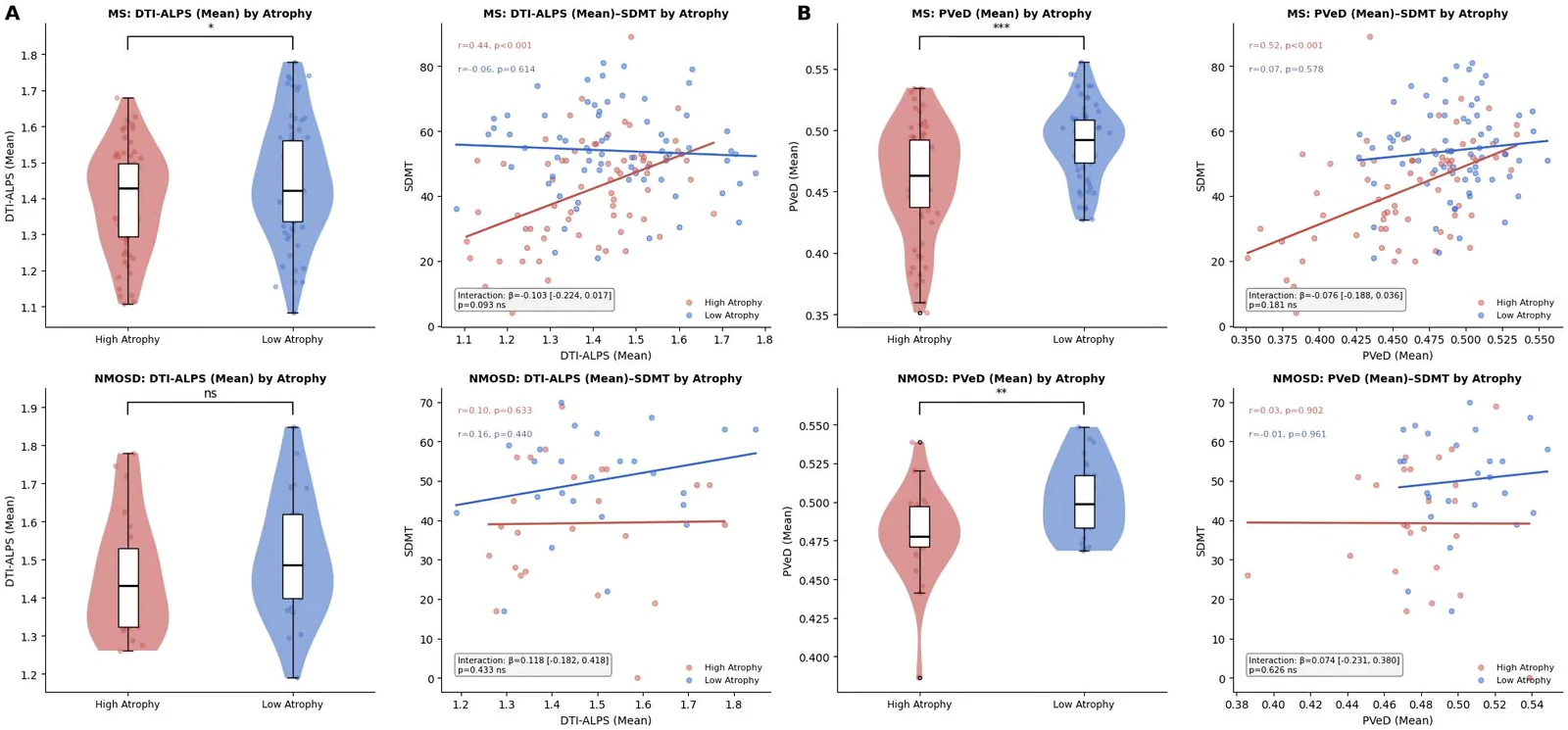
Atrophy subgroup analysis of glymphatic-cognitive associations in MS and NMOSD. MS and NMOSD patients were stratified into high and low subcortical atrophy subgroups based on the median composite score of 14 bilateral subcortical structures. **(A)** DTI-ALPS-based analysis. **(B)** PVeD-based analysis. For each panel, the left column shows the distribution of the respective glymphatic metric between atrophy subgroups (violin plots with embedded box plots and individual data points), and the right column shows scatter plots of the glymphatic metric versus SDMT with Spearman correlation coefficients reported separately for each subgroup. The boxed annotation in each scatter plot reports the result of a formal interaction test (metric × atrophy status, adjusted for age and sex) assessing whether the association between the glymphatic metric and SDMT differed significantly by atrophy status. Upper row: MS; lower row: NMOSD. Subcortical atrophy composite score was derived by z-scoring and averaging volumes of 14 bilateral subcortical structures. High Atrophy = below-median composite score; Low Atrophy = above-median composite score. *p < 0.05; **p < 0.01; ***p < 0.001. SDMT, Symbol Digit Modalities Test; MS, multiple sclerosis; NMOSD, neuromyelitis optica spectrum disorder.

In contrast, in NMOSD, while PVeD was significantly lower in the high atrophy subgroup compared to the low atrophy subgroup (p < 0.01), DTI-ALPS did not differ significantly between atrophy subgroups (p > 0.05). Neither PVeD nor DTI-ALPS showed significant associations with SDMT in either atrophy subgroup in NMOSD (all p > 0.4) and formal interaction tests were similarly non-significant for both PVeD (p = 0.626) and DTI-ALPS (p = 0.433).

### AQP4 antibody status and glymphatic function in NMOSD

As shown in **Figure 6**, AQP4-seropositive NMOSD patients showed significantly lower DTI-ALPS (Mean) and PVeD (Mean) than HC (both p < 0.001), whereas AQP4-seronegative patients did not differ significantly from HC (DTI-ALPS: p = 0.205; PVeD: p = 0.201). Direct comparison between AQP4-seropositive and AQP4-seronegative patients showed no significant difference in DTI-ALPS under either the base covariate model (age, sex, scanner; p = 0.104) or the extended model additionally adjusting for EDSS and disease duration (p = 0.087). PVeD similarly did not differ significantly between the two subgroups under either model (base: p = 0.126; extended: p = 0.211).

**Figure 6:**
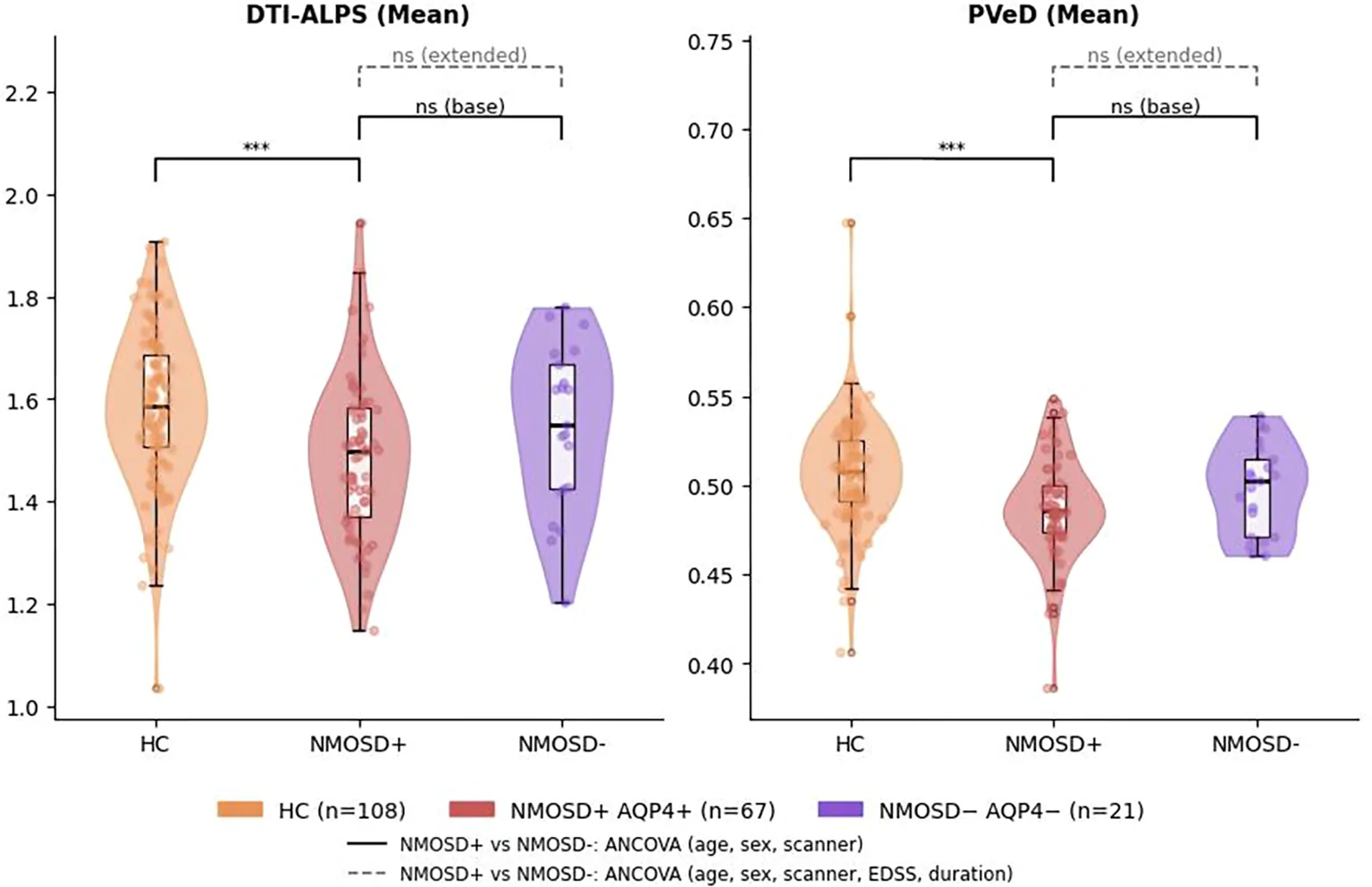
Glymphatic-related indices in NMOSD subgroups stratified by AQP4 antibody status. Violin plots showing DTI-ALPS (Mean; left) and PVeD (Mean; right) in healthy controls (HC), AQP4-seropositive NMOSD (NMOSD+), and AQP4-seronegative NMOSD (NMOSD-) in the combined Siemens and GE cohorts. Box plots indicate median and interquartile range; individual data points are overlaid. Solid brackets denote pairwise group comparisons using ANCOVA adjusted for age, sex, and scanner; for the NMOSD+ versus NMOSD- comparison, the dashed bracket additionally shows results after further adjustment for EDSS and disease duration ("extended"). ***p < 0.001; ns, not significant. HC, healthy controls; NMOSD+, AQP4-IgG seropositive; NMOSD-, AQP4-IgG seronegative.

## Discussion

In this study, we characterized disease-specific patterns of glymphatic-related dysfunction in MS and NMOSD using both DTI-ALPS and PVeD across two independent cohorts. Three principal findings emerged. First, MS and NMOSD exhibited distinct impairment patterns: in MS, glymphatic-related dysfunction was coupled with widespread structural neurodegeneration, white matter lesion burden, and cognitive impairment, whereas in NMOSD, significant associations were restricted to ventricular enlargement and choroid plexus volumes. Second, a parallel multiple mediation model showed that subcortical gray matter atrophy, rather than lesion volume, was the primary independent pathway linking glymphatic-related dysfunction to cognitive impairment in MS, with this association observed specifically in patients with greater atrophy. Third, compared with HC, AQP4-seropositive but not AQP4-seronegative NMOSD patients showed significantly lower PVeD and DTI-ALPS; however, direct comparison between AQP4-seropositive and AQP4-seronegative patients did not reach significance for either metric, even after further adjusting for disease severity, suggesting that this asymmetric pattern relative to HC does not, on its own, establish a robust AQP4-specific effect on either glymphatic-related metric. Across these comparisons, PVeD showed larger effect sizes than DTI-ALPS on average, supporting its potential as a complementary glymphatic-related marker.

Previous systematic reviews and meta-analyses have confirmed that DTI-ALPS indices are significantly lower in MS patients than in HC and are negatively correlated with disease duration, EDSS, and white matter lesion volume (7). Furthermore, patients with greater disability showed lower ALPS indices, associated with more severe brain atrophy, lesion burden, and longer disease duration (8). However, when directly comparing MS and NMOSD, Kim et al. found no significant differences between groups in either DTI-ALPS or free-water indices using Bayesian hypothesis testing (16). Several study characteristics differed from our study, including cohort size, number of scanner platforms, AQP4 serostatus composition, age distribution between disease groups, and statistical framework (Bayesian vs. Our frequentist approach). Notably, our cross-platform harmonization analysis showed that effect sizes changed minimally after ComBat correction (mean absolute change in Cohen’s d = 0.009), indicating that our findings are not attributable to scanner-specific artifacts. Given these differences in design and analytical approach, the discrepancy between the two studies likely reflects methodological factors rather than a fundamental disagreement about underlying biology.

This is consistent with recent methodological concerns that the accuracy of the ALPS index is highly dependent on predefined small ROIs and susceptible to confounding by white matter fiber orientation and anisotropy (17). In our own cohort, manual ROI placement for DTI-ALPS yielded only moderate inter-rater reliability, consistent with a previously reported ICC of 0.599 in native-space manual delineation (23),suggesting that inter-rater variability may attenuate DTI-ALPS sensitivity even when cross-scanner reproducibility is maintained under standardized protocols (24, 25).

In contrast, PVeD is derived through a fully automated periventricular delineation strategy independent of fiber geometry (19). In periventricular white matter, perivascular spaces run largely orthogonal to dominant fiber tracts, enabling the transverse tensor ratio to capture diffusivity components more closely reflecting perivascular fluid movement. Chen et al. demonstrated that lower PVeD was associated with greater amyloid burden and cognitive impairment in the Alzheimer’s disease continuum, interpreting this as a marker of perivenous glymphatic-related fluid dynamics (19). In our cohort, PVeD showed larger effect sizes than DTI-ALPS across all pairwise comparisons, and its structural associations were largely preserved after MD adjustment, whereas DTI-ALPS associations were substantially attenuated, supporting the greater sensitivity of PVeD to glymphatic-related diffusivity relative to DTI-ALPS. It should be noted that our primary PVeD analyses did not exclude visible lesion voxels from the periventricular region of interest; because periventricular white matter in MS may contain demyelinating lesions, PVeD as computed here could in principle reflect lesion-related demyelination and axonal injury in addition to, or instead of, perivascular fluid movement. The separate lesion exclusion sensitivity analysis in our study indicated that the primary PVeD findings, with the exception of the PVeD Left HC-versus-NMOSD comparison, were largely robust to this consideration, suggesting that lesion-related signal alone is unlikely to fully account for our primary results.

The cross-cohort discrepancy in DTI-ALPS sensitivity for NMOSD warrants consideration. AQP4-seropositive proportions were similar between cohorts (Siemens 77.6% vs. GE 74.4%), making this an unlikely explanation. Instead, this discrepancy more likely reflects differences in MRI acquisition protocol (DKI vs. conventional DTI, resolution, direction count), which persisted after ComBat harmonization and are consistent with DTI-ALPS’s known sensitivity to acquisition parameters. In contrast, PVeD demonstrated consistent group differences and larger effect sizes than DTI-ALPS across both cohorts, supporting its greater robustness across platforms.

In the MS group, PVeD showed extensive associations with structural neuroimaging measures, including subcortical gray matter atrophy, white matter lesion burden, and cognitive function, consistent with previously reported associations between DTI-ALPS and neurodegeneration and clinical disability in MS (26). We also observed significant negative correlations between PVeD and choroid plexus volume, such that lower PVeD was associated with larger choroid plexus volume, a direction consistent with the association reported by Preziosa et al. between choroid plexus enlargement and lower DTI-ALPS (27). That study further demonstrated that DTI-ALPS partially mediated the association between choroid plexus enlargement and structural brain damage, including white matter lesions, thalamic atrophy, and cortical atrophy, with relative mediation effects ranging from 25.2% to 52.7%, suggesting that choroid plexus inflammation may contribute to the accumulation of brain damage, at least in part through glymphatic-related dysfunction.

Building on these findings, the mediation analyses in the present study identified subcortical gray matter atrophy as the primary independent structural pathway statistically linking glymphatic-related dysfunction to cognitive impairment (Proportion Mediated = 80.6% in the parallel multiple mediation model), whereas the contribution of lesion volume was not statistically independent of this pathway. Prior work has shown that deep gray matter atrophy partially mediates the relationship between DTI-ALPS and clinical disability and disease duration in relapsing-remitting MS (28), and that in pediatric MS, significant reductions in DTI-ALPS were observed specifically in patients with cognitive impairment, suggesting that glymphatic-related dysfunction may contribute to cognitive decline even in the early stages of disease (29). A recent narrative review further highlighted that impaired glymphatic clearance in MS may promote the accumulation of neuroinflammatory mediators and neurotoxic products, potentially contributing to demyelination and neuronal loss (30).

To further corroborate these mediation findings, MS and NMOSD patients were stratified into high and low subcortical atrophy subgroups. In MS, glymphatic-related metrics were significantly lower in the high atrophy subgroup, and both PVeD and DTI-ALPS were significantly associated with SDMT exclusively in the high atrophy subgroup, with no significant associations observed in the low atrophy subgroup. This pattern is consistent with, although not statistically confirmed by formal interaction testing, the possibility that the relationship between glymphatic-related dysfunction and cognitive impairment in MS may be more pronounced in the presence of subcortical neurodegeneration. In NMOSD, while PVeD was significantly lower in the high atrophy subgroup, neither PVeD nor DTI-ALPS showed significant associations with SDMT in either atrophy subgroup, indicating that even when glymphatic dysfunction co-occurs with subcortical atrophy in NMOSD, it does not translate into cognitive impairment. This dissociation underscores the disease-specific nature of glymphatic-related structural-cognitive coupling in MS versus NMOSD.

In contrast to MS, significant associations in the NMOSD group were restricted to PVeD correlations with ventricular and choroid plexus volumes, with no significant associations observed for subcortical gray matter, lesion volume, or cognitive measures, and neither mediation model reached significance. This restricted pattern is consistent with the distinct immunopathology of NMOSD, in which AQP4 antibody-mediated astrocytopathy primarily causes focal periventricular damage rather than the widespread neurodegeneration characteristic of MS. The exploratory subgroup analysis further revealed that glymphatic reductions were specific to AQP4-seropositive NMOSD patients, whereas seronegative patients did not differ significantly from HC. This pattern relative to HC is consistent with, but does not on its own confirm, a role for AQP4-mediated astrocyte injury in periventricular glymphatic-related impairment. These findings are further supported by prior reports linking enlarged perivascular spaces and choroid plexus enlargement in NMOSD to AQP4 antibody titers and periventricular structural remodeling (9, 10), collectively suggesting that glymphatic involvement in NMOSD operates at the level of periventricular structures rather than widespread parenchymal damage.

## Limitation

Several limitations of the present study should be acknowledged. First, the cross-sectional design precludes the determination of temporal and causal relationships between glymphatic-related dysfunction and structural brain damage; longitudinal studies are needed to establish the directionality of these associations. Second, the AQP4-seronegative subgroup was small, resulting in insufficient statistical power, and the subgroup analysis findings should therefore be interpreted with caution. Third, both DTI-ALPS and PVeD are indirect diffusion MRI-based surrogates that cannot directly quantify CSF-ISF flow; validation against established glymphatic-related reference standards, such as intrathecal contrast-enhanced MRI, remains to be conducted, particularly for PVeD whose application in inflammatory demyelinating diseases has not previously been validated. Fourth, although lesion exclusion sensitivity analysis supported the robustness of PVeD Mean and PVeD Right to lesion-related confounding, the HC-versus-NMOSD difference in PVeD Left was attenuated to non-significance after lesion exclusion in both cohorts, suggesting that this specific comparison may be partly influenced by lesion-related diffusivity changes in the left periventricular white matter and should be interpreted with caution. Besides, cognitive assessments were not available in the GE replication cohort, and the association between PVeD and cognitive performance could therefore not be validated in a second, independent cohort; prospective studies with cognitive data in both cohorts are needed to confirm the reproducibility of this finding.

## Conclusion

In conclusion, MS and NMOSD exhibit distinct disease-specific patterns of glymphatic-related dysfunction. In MS, glymphatic-related impairment is coupled with widespread structural neurodegeneration and cognitive decline, primarily through a subcortical gray matter atrophy pathway. In NMOSD, it is largely restricted to ventricular and choroid plexus volume, without the widespread neurodegenerative coupling observed in MS; although both DTI-ALPS and PVeD were reduced in AQP4-seropositive relative to HC, direct comparison between seropositive and seronegative patients did not confirm a robust AQP4-specific effect for either metric. Across both cohorts, PVeD showed greater overall sensitivity than DTI-ALPS in capturing these disease-specific differences, suggesting its potential as a complementary glymphatic-related marker in inflammatory demyelinating disease.

## Data Availability

The datasets presented in this article are not readily available because the informed consent obtained from participants did not cover public sharing of the data with third parties; sharing outside the original research team would require additional ethical approval and/or renewed participant consent. Requests to access the datasets should be directed to lymzhang70@163.com.
