# Supplementary material for: Disease-specific patterns of glymphatic-related dysfunction between multiple sclerosis and neuromyelitis optica spectrum disorder
Source: Front Immunol. 2026 Sep 18;17:1917373. doi: 10.3389/fimmu.2026.1917373 (PMC13630702; doi:10.3389/fimmu.2026.1917373)
Supplement: Supplementary file 1 [file DataSheet1.pdf]

Supplementary Methods

MRI data processing and analysis

DTI processing

Calculation of DTI-ALPS

DTI data preprocessing was performed using FSL and MRtrix3. For the Siemens cohort, DTI images were extracted from the DKI sequence using b = 0 and b = 1000 s/mm^2 volumes. The preprocessing pipeline included dwidenoise, mrdegibbs, eddy_correct, dwibiascorrect, dwitensor, and tensor2metricd[1]. Diffusion tensor fitting was performed using FSL DTIFIT to generate voxel-wise Dxx, Dyy, and Dzz maps, which were nonlinearly registered to MNI standard space using ANTs. The same preprocessing pipeline was applied to the GE cohort DTI data.

Four ROIs were manually delineated bilaterally in the projection fiber area and association fiber area for each participant, and the mean Dxx, Dyy, and Dzz values were extracted for each ROI. The DTI-ALPS index was calculated as:[2]

ALPS index = mean (Dxxproj, Dxxassoci)/ mean (Dyyproj, Dzzassoci)

where Dxx_proj and Dxx_assoc denote x-axis diffusivity in the projection and association fiber ROIs, respectively; Dyy_proj denotes y-axis diffusivity in the projection fiber ROI; and Dzz_assoc denotes z-axis diffusivity in the association fiber ROI. Left and right DTI-ALPS indices were calculated separately. Inter-rater reliability of ROI placement was assessed by two independent raters (ZK and DX in the Siemens cohort; CX and PY in the GE cohort) using intraclass correlation coefficients (ICC).

Calculation of PVeD

PVeD was computed following the framework proposed by Chen et al.[3], which was

developed as an automated, anatomy-guided metric to overcome the region-of-interest (ROI) limitations of conventional DTI-ALPS and to capture transverse interstitial fluid (ISF) diffusion along perivascular spaces in the periventricular white matter. The method was adapted for application in demyelinating diseases as follows. First, voxel-wise transverse tensor ratio (TTR) was calculated from the diffusion tensor eigen-components (Dxx, Dyy, and Dzz), under the anatomical assumption that deep medullary veins predominantly run along the left-right (x-axis) direction, such that ISF diffusion within perivascular spaces is expected to be maximal along this transverse axis. TTR was defined as the proportion of x-axis diffusivity relative to the total diffusivity, with higher values indicating greater transverse diffusion dominance. To define the periventricular area (PVeA) in an automated and subject-specific manner, an anatomy-guided region-growing strategy was applied. A white matter (WM) mask was first derived from the mean diffusivity (MD) map to exclude gray matter and cerebrospinal fluid. A lateral ventricle pre-mask defined in MNI space was then nonlinearly registered to each subject's MD space and refined to generate an individualized ventricular mask. Based on anatomical constraints, directional dilation was performed along the transverse ventricular axis, with maximal expansion applied to the ventricular body (where deep medullary veins are densest) and minimal expansion near ventricular margins, yielding an initial PVeA. The initial PVeA was subsequently intersected with the WM mask to obtain the final PVeA, ensuring tissue specificity to periventricular white matter. PVeD was then calculated as the median TTR value across all voxels within the final PVeA, representing the regional transverse diffusivity level. The median was used instead of the mean to reduce sensitivity to outliers, such as focal vascular abnormalities or partial volume effects.

For the lesion exclusion sensitivity analysis, lesion masks segmented in FLAIR space were first registered to native T1 space using ANTs, and then affinely co-registered to native diffusion (b0) space. The b0-space lesion mask was binarized and included as an auxiliary image (--other_image) during q-space diffeomorphic reconstruction (QSDR; DSI Studio, diffusion sampling length ratio = 1.25, output resolution = 2 mm

isotropic), such that it underwent the same nonlinear spatial normalization as the diffusion data and was thereby co-registered into the same standardized TTR space used for PVeD calculation. The normalized lesion mask was then exported from the resulting reconstruction file and used to exclude lesion-overlapping voxels from the periventricular ROI when recalculating PVeD.

1. Tournier JD, Smith R, Raffelt D, et al. MRtrix3: A fast, flexible and open software framework for medical image processing and visualisation. *Neuroimage* 2019; 202: 116137. 2019/09/02. DOI: 10.1016/j.neuroimage.2019.116137.
2. Taoka T, Masutani Y, Kawai H, et al. Evaluation of glymphatic system activity with the diffusion MR technique: diffusion tensor image analysis along the perivascular space (DTI-ALPS) in Alzheimer's disease cases. *Jpn J Radiol* 2017; 35: 172-178. 2017/02/16. DOI: 10.1007/s11604-017-0617-z.
3. Chen CL, Son SJ, Schweitzer N, et al. Periventricular diffusivity reflects APOE epsilon4-modulated amyloid accumulation and cognitive impairment in the Alzheimer's disease continuum. *Alzheimers Dement* 2025; 21: e70659. 2025/09/18. DOI: 10.1002/alz.70659.

Supplementary Table S1. Effect Sizes (Cohen's d) for Between-Group Differences in DTI-ALPS and PVeD

	Siemens Cohort (Primary)			GE Cohort (Replication)			Overall Mean
**Metric**	**HC vs MS**	**HC vs NMOSD**	**MS vs NMOSD**	**HC vs MS**	**HC vs NMOSD**	**MS vs NMOSD**	
**DTI-ALPS (Right)**	0.647	0.364	0.295	1.089	0.620	0.551	0.611
**DTI-ALPS (Left)**	0.528	0.163	0.345	1.206	0.793	0.360	
**DTI-ALPS (Mean)**	0.663	0.288	0.364	1.366	0.851	0.512	
**PVeD (Right)**	**0.789**	**0.535**	**0.372**	**1.111**	0.573	**0.595**	**0.729**
**PVeD (Left)**	**0.885**	**0.581**	**0.446**	**1.215**	0.526	**0.947**	
**PVeD (Mean)**	**0.857**	**0.581**	**0.417**	1.273	0.609	**0.806**	

Cohen's d was calculated as the absolute standardized mean difference between groups. Mean = average of left and right hemispheres. Bold green values indicate larger effect sizes for PVeD compared to DTI-ALPS in the corresponding comparison. HC = healthy controls; MS = multiple sclerosis; NMOSD = neuromyelitis optica spectrum disorder.

Supplementary Table S2 Between-group effect sizes (Cohen's d) for DTI-ALPS and PVeD before and after cross-platform harmonization using ComBat.

Glymphatic-related metric	Cohort	Pairwise comparison	Cohen's d (pre-ComBat)	Cohen's d (post-ComBat)	Δd (post − pre)
DTI-ALPS (Right)	Siemens	HC vs MS	0.647	0.639	-0.009
DTI-ALPS (Right)	Siemens	HC vs NMOSD	0.364	0.359	-0.005
DTI-ALPS (Right)	Siemens	MS vs NMOSD	0.295	0.291	-0.004
DTI-ALPS (Right)	GE	HC vs MS	1.089	1.112	+0.022
DTI-ALPS (Right)	GE	HC vs NMOSD	0.620	0.634	+0.013
DTI-ALPS (Right)	GE	MS vs NMOSD	0.551	0.561	+0.010
DTI-ALPS (Left)	Siemens	HC vs MS	0.528	0.520	-0.008
DTI-ALPS (Left)	Siemens	HC vs NMOSD	0.163	0.158	-0.005
DTI-ALPS (Left)	Siemens	MS vs NMOSD	0.345	0.342	-0.003
DTI-ALPS (Left)	GE	HC vs MS	1.206	1.228	+0.022
DTI-ALPS (Left)	GE	HC vs NMOSD	0.793	0.804	+0.011
DTI-ALPS (Left)	GE	MS vs NMOSD	0.360	0.368	+0.009
DTI-ALPS (Mean)	Siemens	HC vs MS	0.663	0.655	-0.008
DTI-ALPS (Mean)	Siemens	HC vs NMOSD	0.288	0.284	-0.004
DTI-ALPS (Mean)	Siemens	MS vs NMOSD	0.364	0.361	-0.003
DTI-ALPS (Mean)	GE	HC vs MS	1.366	1.387	+0.021
DTI-ALPS (Mean)	GE	HC vs NMOSD	0.851	0.862	+0.012
DTI-ALPS (Mean)	GE	MS vs NMOSD	0.512	0.520	+0.008
PVeD (Right)	Siemens	HC vs MS	0.789	0.785	-0.004
PVeD (Right)	Siemens	HC vs NMOSD	0.535	0.532	-0.002

PVeD (Right)	Siemens	MS vs NMOSD	0.372	0.370	-0.002
PVeD (Right)	GE	HC vs MS	1.111	1.124	+0.013
PVeD (Right)	GE	HC vs NMOSD	0.573	0.580	+0.007
PVeD (Right)	GE	MS vs NMOSD	0.595	0.600	+0.006
PVeD (Left)	Siemens	HC vs MS	0.885	0.874	-0.011
PVeD (Left)	Siemens	HC vs NMOSD	0.581	0.575	-0.006
PVeD (Left)	Siemens	MS vs NMOSD	0.446	0.440	-0.006
PVeD (Left)	GE	HC vs MS	1.215	1.245	+0.030
PVeD (Left)	GE	HC vs NMOSD	0.526	0.540	+0.015
PVeD (Left)	GE	MS vs NMOSD	0.947	0.967	+0.020
PVeD (Mean)	Siemens	HC vs MS	0.857	0.853	-0.005
PVeD (Mean)	Siemens	HC vs NMOSD	0.581	0.578	-0.003
PVeD (Mean)	Siemens	MS vs NMOSD	0.417	0.415	-0.002
PVeD (Mean)	GE	HC vs MS	1.273	1.287	+0.014
PVeD (Mean)	GE	HC vs NMOSD	0.609	0.617	+0.007
PVeD (Mean)	GE	MS vs NMOSD	0.806	0.813	+0.007

Note: ComBat harmonization was applied with scanner (Siemens/GE) specified as the batch variable, and diagnostic group, age, and sex protected as biological covariates (Siemens n = 254, GE n = 138). Effect sizes were recomputed within each cohort using the harmonized values and compared against the unharmonized (pre-ComBat) effect sizes. Across all 36 pairwise comparisons, the mean absolute change in Cohen's d was 0.015 (range: 0.002–0.041), indicating that between-group effect sizes were minimally affected by cross-platform harmonization. HC = healthy controls; MS = multiple sclerosis; NMOSD = neuromyelitis optica spectrum disorder; DTI-ALPS = Diffusion Tensor Image Analysis Along the Perivascular Space; PVeD = periventricular diffusivity. Positive Cohen's d values indicate higher values in the first-named group of each comparison (e.g., HC > MS for a positive HC vs MS value); negative values indicate the reverse. Δd = difference between post- and pre-ComBat effect sizes; values close to zero indicate that the effect size was robust to harmonization.

Table S3. Effect Sizes (Cohen's d) and Significance for PVeD Before vs After Lesion Exclusion

	Siemens Cohort (Primary; MS n=138, NMOSD n=49)						GE Cohort (Replication; MS n=44, NMOSD n=36)					
	HC vs MS		HC vs NMOSD		MS vs NMOSD		HC vs MS		HC vs NMOSD		MS vs NMOSD	
**Metric**	**Before**	**After**	**Before**	**After**	**Before**	**After**	**Before**	**After**	**Before**	**After**	**Before**	**After**
**PVeD (Right)**	0.807***	**1.181*** **	0.535**	**0.973*** **	0.393*	**0.433* **	1.157***	**1.565*** **	0.567*	**0.861*** **	0.627**	**0.863*** **
	p=<0.001	*p=<0.001*	*p=0.008*	*p=<0.001*	*p=0.019*	*p=0.010*	*p=<0.001*	*p=<0.001*	*p=0.010*	*p=<0.001*	*p=0.007*	*p=<0.001*
**PVeD (Left)**	0.897***	0.471**	0.581**	**0.112**	0.455**	0.379*	1.228***	0.798***	0.550*	**0.287**	0.922***	0.589*
	p=<0.001	*p=0.004*	*p=0.004*	*p=0.571*	*p=0.007*	*p=0.024*	*p=<0.001*	*p=<0.001*	*p=0.013*	*p=0.187*	*p=<0.001*	*p=0.011*
**PVeD (Mean)**	0.884***	0.841***	0.582**	0.565**	0.441**	0.416*	1.290***	1.228***	0.598**	0.575**	0.824***	0.760**
	p=<0.001	*p=<0.001*	*p=0.004*	*p=0.005*	*p=0.009*	*p=0.013*	*p=<0.001*	*p=<0.001*	*p=0.007*	*p=0.009*	*p=<0.001*	*p=0.001*

Cohen's d values are absolute standardized mean differences; asterisks denote significance of the corresponding pairwise t-test (*p<0.05, **p<0.01, ***p<0.001; ns = not significant). Small italic values below each d indicate the exact p-value. Bold green values indicate a larger effect size after lesion exclusion compared to before. Bold red values with light red shading indicate comparisons that were significant before lesion exclusion but became non-significant after (i.e., a change in significance pattern). This sensitivity analysis was restricted to participants with adequate lesion/FLAIR image quality for lesion masking; compared to the full analytic sample (Supplementary Table S1), MS participants were excluded in both cohorts (Siemens: n=138 vs. 150; GE: n=44 vs. 46) and NMOSD participants were excluded in the GE cohort (n=36 vs. 39) due to insufficient lesion/FLAIR image quality; all Siemens NMOSD participants (n=49) had adequate image quality and were included. "PVeD (Total)" refers to a composite PVeD value provided in the original dataset and is distinct from the bilateral-mean "PVeD (Mean)" reported in Supplementary Table S1. HC = healthy controls; MS = multiple sclerosis; NMOSD = neuromyelitis optica spectrum disorder.

Table S4. Sensitivity Analysis: Partial Correlations Before and After Adjustment for Mean Diffusivity (Mean Values)

Metric	Group	Outcome	r (before MD)	p (before MD)	r (after MD)	p (after MD)	Still significant
DTI-ALPS (Mean)	MS — Siemens	LV-L	-0.31	<0.001	-0.139	0.252	No
DTI-ALPS (Mean)	MS — Siemens	LV-R	-0.278	0.001	-0.104	0.403	No
DTI-ALPS (Mean)	MS — Siemens	3rd-Vent	-0.195	0.028	0.042	0.704	No
DTI-ALPS (Mean)	MS — Siemens	CSF	-0.191	0.031	-0.025	0.790	No
DTI-ALPS (Mean)	MS — Siemens	ChP-R	-0.239	0.006	-0.06	0.641	No
DTI-ALPS (Mean)	MS — Siemens	ChP-L	-0.264	0.002	-0.109	0.403	No
DTI-ALPS (Mean)	MS — Siemens	Thal-L	0.323	<0.001	0.218	0.062	No
DTI-ALPS (Mean)	MS — Siemens	Thal-R	0.314	<0.001	0.196	0.092	No
DTI-ALPS (Mean)	MS — Siemens	Caud-R	0.202	0.023	0.08	0.499	No
DTI-ALPS (Mean)	MS — Siemens	Put-L	0.375	<0.001	0.238	0.058	No
DTI-ALPS (Mean)	MS — Siemens	Put-R	0.304	<0.001	0.182	0.109	No
DTI-ALPS (Mean)	MS — Siemens	Pall-L	0.321	<0.001	0.196	0.092	No
DTI-ALPS (Mean)	MS — Siemens	Pall-R	0.184	0.037	0.122	0.324	No
DTI-ALPS (Mean)	MS — Siemens	Hipp-L	0.248	0.005	0.225	0.062	No
DTI-ALPS (Mean)	MS — Siemens	Amyg-L	0.266	0.002	0.19	0.095	No
DTI-ALPS (Mean)	MS — Siemens	Acc-L	0.233	0.008	0.093	0.447	No
DTI-ALPS (Mean)	MS — Siemens	Acc-R	0.217	0.013	0.103	0.403	No
DTI-ALPS (Mean)	MS — Siemens	WMVol	0.355	<0.001	0.278	0.020	Yes
DTI-ALPS (Mean)	MS — Siemens	LesionV	-0.262	0.003	-0.036	0.704	No
DTI-ALPS (Mean)	MS — Siemens	HAMD	-0.182	0.038	-0.163	0.170	No
DTI-ALPS (Mean)	MS — Siemens	MoCA	0.173	0.049	0.072	0.550	No
PVeD (Mean)	MS — Siemens	LV-L	-0.793	<0.001	-0.73	<0.001	Yes

PVeD (Mean)	MS — Siemens	LV-R	-0.83	<0.001	-0.765	<0.001	**Yes**
PVeD (Mean)	MS — Siemens	3rd-Vent	-0.597	<0.001	-0.461	<0.001	**Yes**
PVeD (Mean)	MS — Siemens	CSF	-0.54	<0.001	-0.431	<0.001	**Yes**
PVeD (Mean)	MS — Siemens	ChP-R	-0.532	<0.001	-0.419	<0.001	**Yes**
PVeD (Mean)	MS — Siemens	ChP-L	-0.686	<0.001	-0.6	<0.001	**Yes**
PVeD (Mean)	MS — Siemens	Thal-L	0.487	<0.001	0.438	<0.001	**Yes**
PVeD (Mean)	MS — Siemens	Thal-R	0.506	<0.001	0.428	<0.001	**Yes**
PVeD (Mean)	MS — Siemens	Caud-L	0.344	<0.001	0.243	0.005	**Yes**
PVeD (Mean)	MS — Siemens	Caud-R	0.262	0.003	0.154	0.078	**No**
PVeD (Mean)	MS — Siemens	Put-L	0.513	<0.001	0.379	<0.001	**Yes**
PVeD (Mean)	MS — Siemens	Put-R	0.488	<0.001	0.413	<0.001	**Yes**
PVeD (Mean)	MS — Siemens	Pall-L	0.311	<0.001	0.186	0.036	**Yes**
PVeD (Mean)	MS — Siemens	Hipp-L	0.397	<0.001	0.358	<0.001	**Yes**
PVeD (Mean)	MS — Siemens	Hipp-R	0.438	<0.001	0.391	<0.001	**Yes**
PVeD (Mean)	MS — Siemens	Amyg-L	0.244	0.005	0.153	0.078	**No**
PVeD (Mean)	MS — Siemens	Acc-L	0.439	<0.001	0.35	<0.001	**Yes**
PVeD (Mean)	MS — Siemens	Acc-R	0.465	<0.001	0.384	<0.001	**Yes**
PVeD (Mean)	MS — Siemens	CT-L	0.317	<0.001	0.236	0.007	**Yes**
PVeD (Mean)	MS — Siemens	CT-R	0.231	0.008	0.163	0.066	**No**
PVeD (Mean)	MS — Siemens	WMVol	0.383	<0.001	0.326	<0.001	**Yes**
PVeD (Mean)	MS — Siemens	GMVol	0.187	0.035	0.188	0.036	**Yes**
PVeD (Mean)	MS — Siemens	LesionV	-0.512	<0.001	-0.361	<0.001	**Yes**
PVeD (Mean)	MS — Siemens	HAMD	-0.202	0.022	-0.166	0.064	**No**
PVeD (Mean)	MS — Siemens	SDMT	0.287	<0.001	0.206	0.020	**Yes**
PVeD (Mean)	MS — Siemens	MoCA	0.177	0.043	0.133	0.129	**No**

PVeD (Mean)	NMOSD — Siemens	LV-L	-0.526	0.005	-0.537	0.002	**Yes**
PVeD (Mean)	NMOSD — Siemens	LV-R	-0.517	0.005	-0.527	0.002	**Yes**
PVeD (Mean)	NMOSD — Siemens	ChP-L	-0.428	0.047	-0.44	0.017	**Yes**
PVeD (Mean)	MS — GE	LV-L	-0.767	<0.001	-0.723	<0.001	**Yes**
PVeD (Mean)	MS — GE	LV-R	-0.769	<0.001	-0.693	<0.001	**Yes**
PVeD (Mean)	MS — GE	3rd-Vent	-0.61	<0.001	-0.475	0.007	**Yes**
PVeD (Mean)	MS — GE	CSF	-0.554	<0.001	-0.403	0.025	**Yes**
PVeD (Mean)	MS — GE	ChP-R	-0.498	0.004	-0.458	0.008	**Yes**
PVeD (Mean)	MS — GE	ChP-L	-0.611	<0.001	-0.542	0.001	**Yes**
PVeD (Mean)	MS — GE	Thal-R	0.392	0.049	0.26	0.158	**No**
PVeD (Mean)	MS — GE	CT-L	0.475	0.007	0.402	0.025	**Yes**
PVeD (Mean)	MS — GE	CT-R	0.44	0.017	0.337	0.085	**No**
PVeD (Mean)	NMOSD — GE	LV-L	-0.604	0.001	-0.573	0.002	**Yes**
PVeD (Mean)	NMOSD — GE	LV-R	-0.612	0.001	-0.562	0.002	**Yes**
PVeD (Mean)	NMOSD — GE	3rd-Vent	-0.496	0.018	-0.484	0.014	**Yes**
PVeD (Mean)	NMOSD — GE	CSF	-0.578	0.002	-0.56	0.002	**Yes**
PVeD (Mean)	NMOSD — GE	ChP-R	-0.489	0.018	-0.419	0.049	**Yes**
PVeD (Mean)	NMOSD — GE	ChP-L	-0.485	0.018	-0.391	0.071	**No**

Only correlations significant before MD adjustment (FDR-adjusted p < 0.05, age- and sex-adjusted) are listed. 'r (after MD)' and 'p (after MD)' reflect partial correlations additionally adjusting for mean diffusivity (MD_mean, the average of md_r_d and md_l_d). Bold green indicates the correlation remained significant after MD adjustment; bold red with light red shading indicates the correlation was no longer significant, suggesting the original association may be substantially explained by MD rather than glymphatic-specific diffusivity.

Table S5. Domain Specificity of the PVeD-Cognition Association (Siemens MS Cohort)

**A. Partial correlations between PVeD and cognitive measures**				
**Comparison**	**r**	**p**	**n**	**Covariates**
PVeD vs SDMT	0.287	<0.001	150	age + gender
PVeD vs SDMT	0.271	<0.001	150	age + gender + EDSS
PVeD vs DST	0.039	0.633	150	age + gender
PVeD vs MMSE	0.043	0.598	150	age + gender
PVeD vs MoCA	0.177	0.030	150	age + gender

**B. Steiger's Z tests comparing PVeD-SDMT correlation strength against other cognitive domains**			
**Comparison**	**z**	**p**	**Significant (p<0.05)**
**PVeD-SDMT vs PVeD-DST**	**2.61**	**0.009**	**Yes**
**PVeD-SDMT vs PVeD-MMSE**	**2.68**	**0.007**	**Yes**
PVeD-SDMT vs PVeD-MoCA	1.36	0.173	No

Partial correlations (Spearman) were computed after regressing out the listed covariates from both PVeD and the cognitive measure. Steiger's Z tests compare dependent (overlapping) correlations within the same sample, testing whether the PVeD-SDMT association is significantly stronger than PVeD's association with each other cognitive measure. Bold green rows indicate a statistically significant difference (p<0.05). DST = Digit Span Test; MMSE = Mini-Mental State Examination; MoCA = Montreal Cognitive Assessment; SDMT = Symbol Digit Modalities Test; EDSS = Expanded Disability Status Scale.

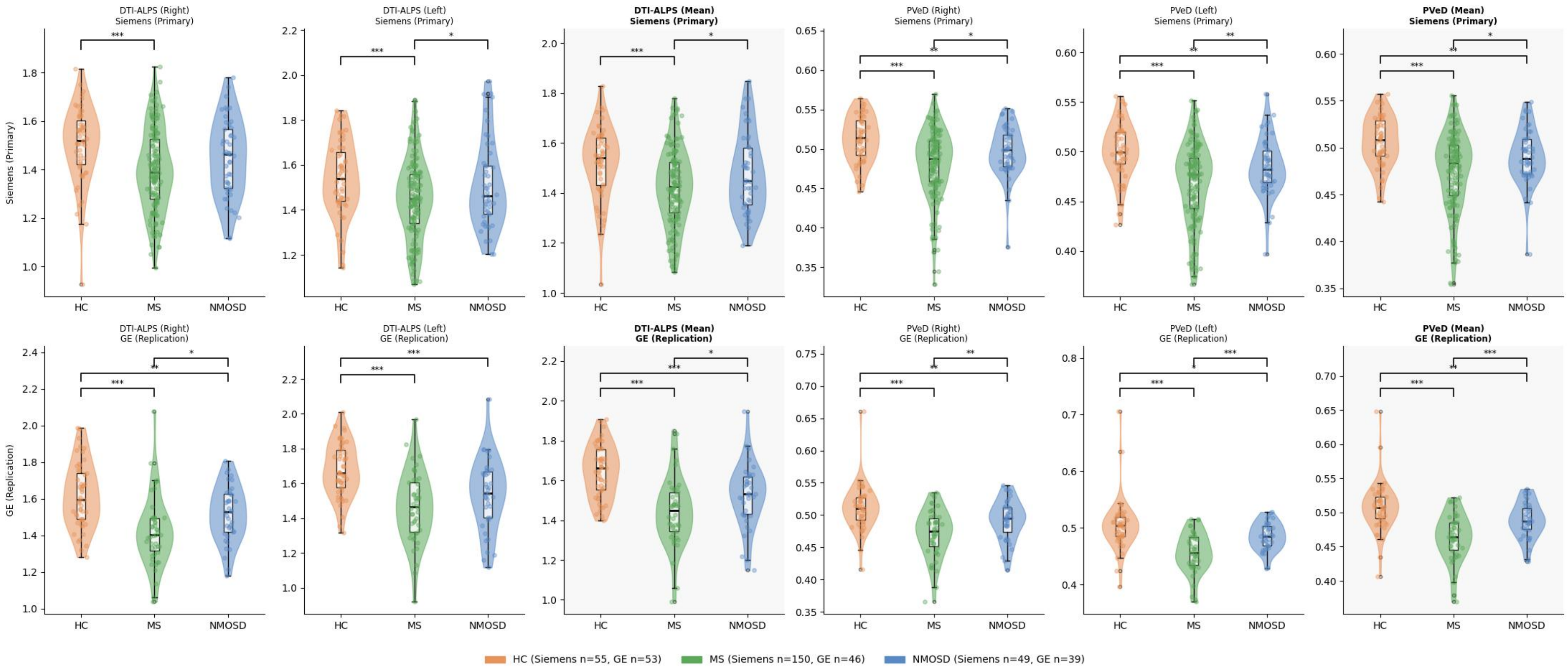

Supplementary Figure S1. Group comparisons of DTI-ALPS and PVeD indices across HC, MS, and NMOSD. Violin plots with embedded box plots and individual data points show the distribution of DTI-ALPS (Right, Left and Mean) and PVeD (Right, Left and Mean) in the Siemens primary cohort (top row) and GE replication cohort (bottom row). Box plots indicate the median and interquartile range; whiskers extend to 1.5 times the interquartile range. Between-group differences were assessed using ANCOVA with age and sex as covariates. Significance brackets indicate post-hoc pairwise comparisons: *$p < 0.05$, **$p < 0.01$, ***$p < 0.001$. HC = healthy controls; MS = multiple sclerosis; NMOSD = neuromyelitis optica spectrum disorder

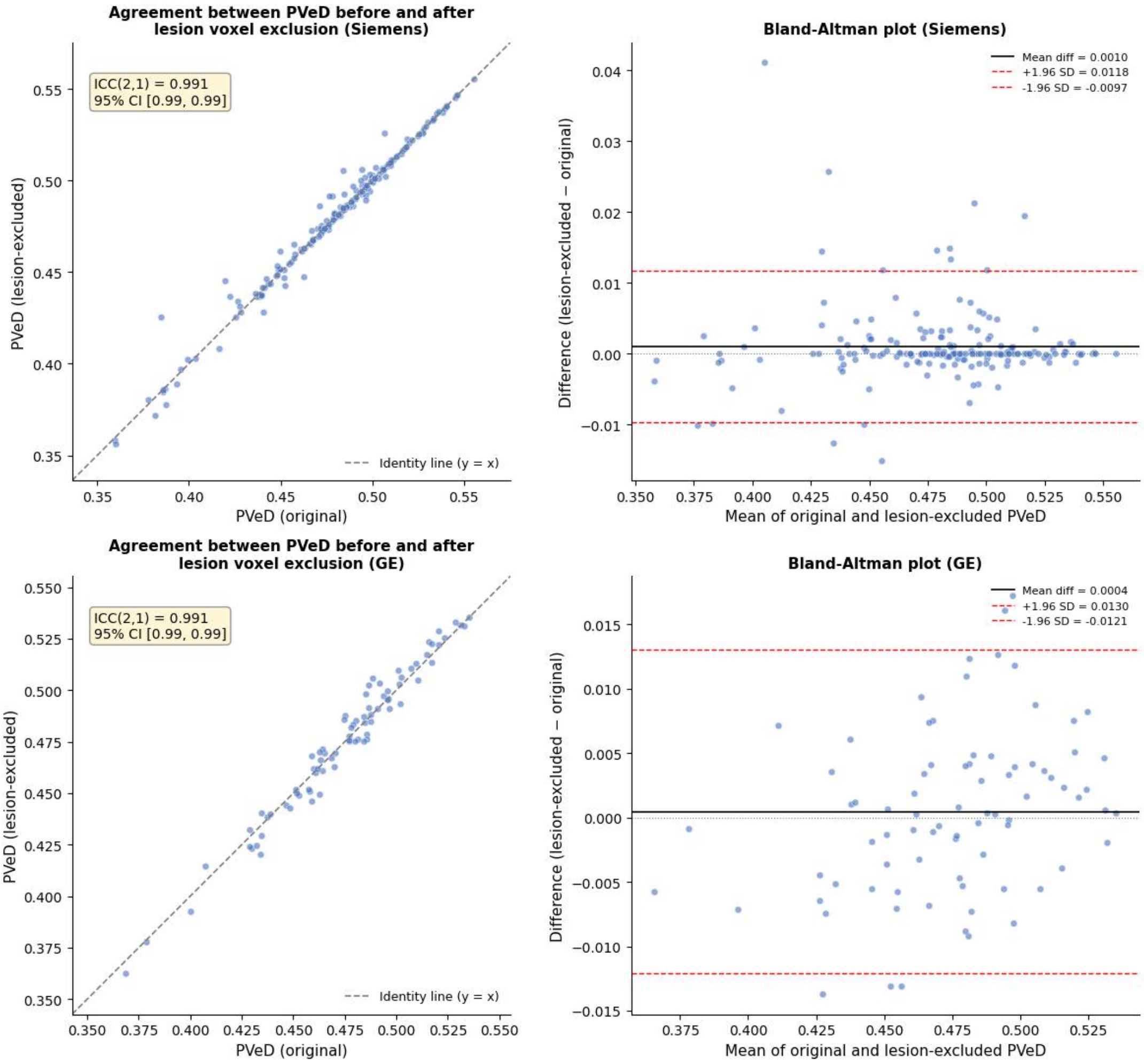

Supplementary Figure S2. Agreement between PVeD values before and after periventricular lesion exclusion. Left column: scatter plots comparing PVeD (total) values calculated before (x-axis) and after (y-axis) excluding visible periventricular lesion voxels from the region of interest, in the Siemens (top) and GE (bottom) cohorts. The dashed line indicates perfect agreement (identity line, y = x). Intraclass correlation coefficients (ICC(2,1)) with 95% confidence intervals are shown in each panel. Right column: corresponding Bland-Altman plots showing the difference between lesion-excluded and original PVeD values (y-axis) against their mean (x-axis). The solid black line indicates the mean difference; dashed red lines indicate the 95% limits of agreement (mean difference ± 1.96 SD). Both cohorts showed high agreement between PVeD values calculated before and after lesion exclusion (ICC ≥ 0.99).

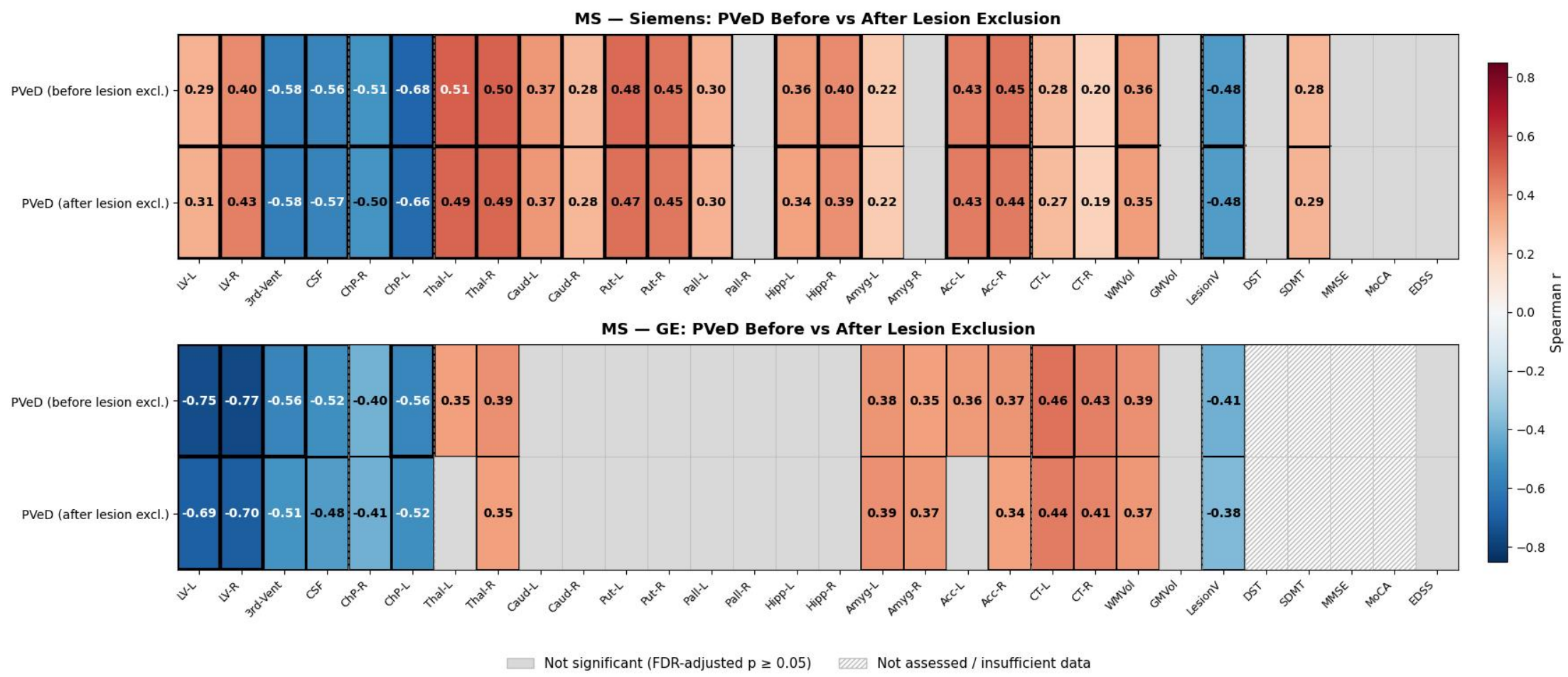

Supplementary Figure S3. Partial correlations of PVeD with structural and clinical measures before and after lesion exclusion in MS. Heatmaps showing partial correlations (Spearman r, adjusted for age and sex) between PVeD and structural neuroimaging and clinical measures in MS patients, before (top row of each panel) and after (bottom row) excluding visible periventricular lesion voxels from the region of interest, in the Siemens (top panel) and GE (bottom panel) cohorts. Colored cells indicate significant correlations after FDR correction (color scale denotes correlation strength and direction); light grey cells indicate correlations tested but not significant; hatched white cells indicate correlations that could not be assessed due to missing data, including cognitive measures (DST, SDMT, MMSE, MoCA) not collected in the GE cohort. Cell border thickness reflects statistical significance level. The overall pattern and number of significant correlations were highly consistent before and after lesion exclusion in both cohorts, supporting that PVeD's associations with structural and cognitive outcomes are not primarily driven by lesion-related demyelination.

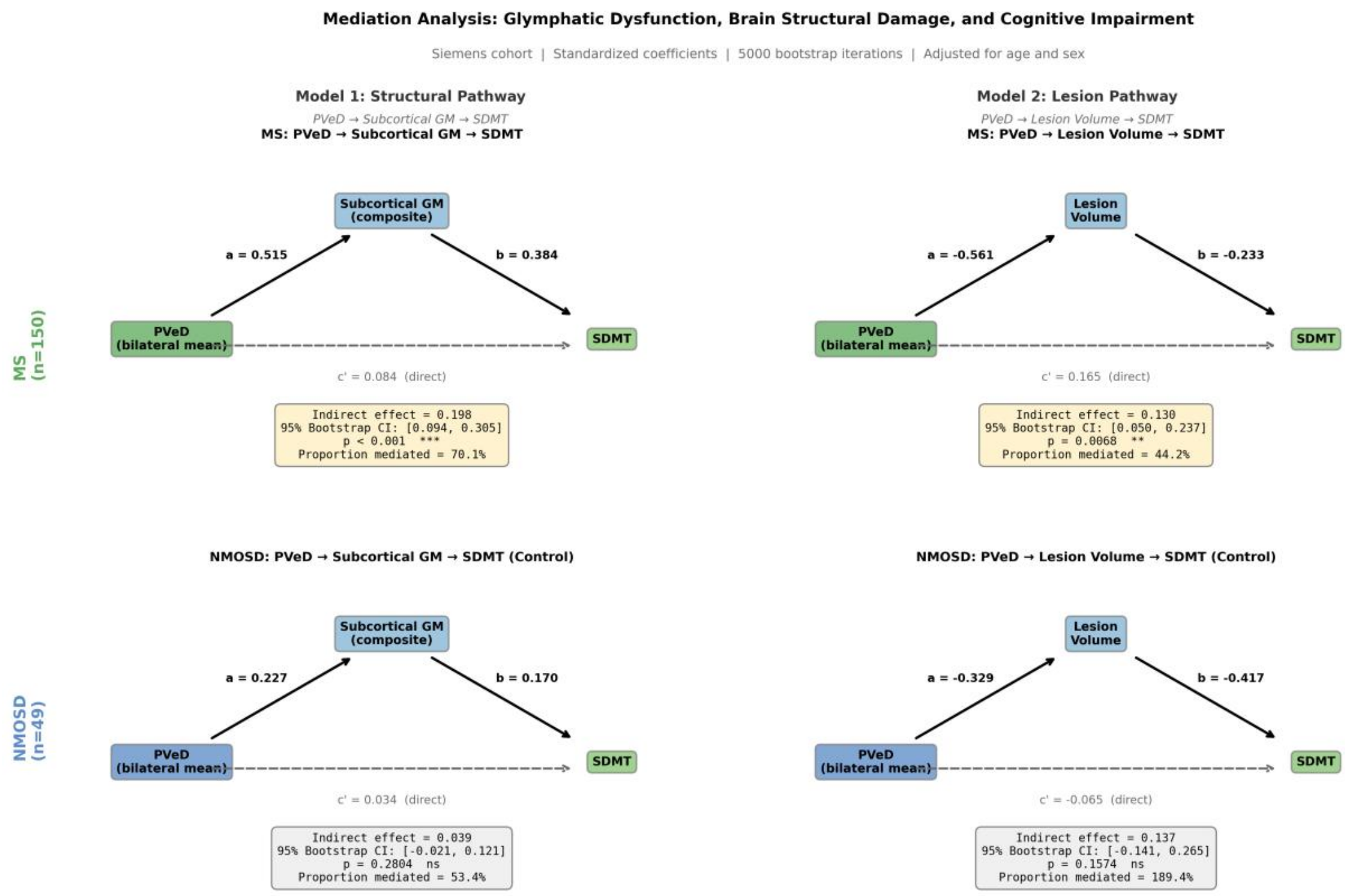

Supplementary Figure S4. Mediation analysis of the association between glymphatic dysfunction, structural brain damage, and cognitive impairment. Path diagrams illustrate two parallel mediation models tested in the MS group (top row) and the NMOSD group (bottom row) as a disease-specific comparison in the Siemens cohort. Model 1 (left column): PVeD (bilateral mean) → subcortical gray matter composite score → SDMT. Model 2 (right column): PVeD (bilateral mean) → white matter lesion volume → SDMT. Path coefficients (a, b, c') represent standardized regression coefficients. Dashed arrows indicate direct effects. Indirect effects were estimated using bias-corrected bootstrap confidence intervals (5000 iterations), adjusted for age and sex. The proportion mediated (PM) represents the ratio of the indirect to the total effect. PM = N/A indicates non-significant mediation. **$p < 0.01$; ***$p < 0.001$; ns = not significant. PVeD = periventricular diffusivity; GM = gray matter; SDMT = Symbol Digit Modalities Test.
